# Arterial tortuosity syndrome in two Italian paediatric patients

**DOI:** 10.1186/1750-1172-4-20

**Published:** 2009-09-25

**Authors:** Marco Ritelli, Bruno Drera, Mariano Vicchio, Giovanni Puppini, Paolo Biban, Mara Pilati, Maria Antonia Prioli, Sergio Barlati, Marina Colombi

**Affiliations:** 1 Division of Biology and Genetics, Department of Biomedical Sciences and Biotechnology, Medical Faculty, University of Brescia, Brescia, Italy; 2 Department of Cardio-Thoracic and Respiratory Sciences, Second University of Naples, Naples, Italy; 3 Department of Radiology, Ospedale Civile Maggiore, Verona, Italy; 4 Department of Pediatry, Ospedale Civile Maggiore, Verona, Italy; 5 Department of Cardiology, Ospedale Civile Maggiore, Verona, Italy

## Abstract

**Background:**

Arterial tortuosity syndrome (ATS) (OMIM #208050) is a rare autosomal recessive connective tissue disorder characterized by tortuosity and elongation of the large and medium-sized arteries, propensity to aneurysms formation, vascular dissection, and pulmonary arteries stenosis. ATS is caused by mutations in *SLC2A10 *gene, encoding for the facilitative glucose transporter 10 (GLUT10). So far, 17 *SLC2A10 *mutations have been reported in 32 families, two of which were Italian with a total of five patients. Here we present the clinical and molecular characterization of two novel Italian paediatric ATS patients.

**Methods:**

The exons and intronic flanking regions of *SLC2A10 *gene were amplified and direct sequencing was performed.

**Results:**

In both patients, the involvement of major- and medium-sized arteries was characteristic; the nonvascular connective tissue manifestations were mild and not pathognomic of the disorder. Both patients, born from non-consanguineous parents, were heterozygous for two different *SLC2A10 *mutations, three of which were recurrent and one was novel (p.Arg231Trp). This mutation is localized at the endofacial loop between the transmembrane domains 6 and 7 of GLUT10.

**Conclusion:**

Two novel ATS patients were characterized at clinical and molecular level. Overall, four ATS unrelated families are known in Italy so far. Though ATS clinical delineation improved in the last years, further works in the comprehension of disease presentation and complications onset, particularly in paediatric age, and on ATS molecular basis are needed to add new insights for diagnosis and prevention strategies for related complications.

## Background

Arterial tortuosity syndrome (ATS) (OMIM #208050) is an extremely rare autosomal recessive disorder characterized by tortuosity and elongation of the large and medium-sized arteries, propensity to aneurysms formation, vascular dissection, and pulmonary arteries stenosis. Other typical manifestations are dysmorphic features, hyperextensible skin, *cutis laxa*, herniae, skeletal abnormalities, joints hypermobility, and congenital contractures [1-4]. ATS is due to mutations in *SLC2A10 *gene, located on chromosome 20q13.1 and encoding for the 541 amino acid facilitative glucose transporter 10 (GLUT10), consisting in 12 transmembrane hydrophobic segments connected by 5 intracellular and 6 extracellular loops [5,6]. So far, 17 *SLC2A10 *mutations have been reported in 32 ATS families [3-5,7,8]. In this work we report the clinical findings and the molecular characterization of two Italian paediatric ATS patients.

## Case presentation

**Figure 1:**
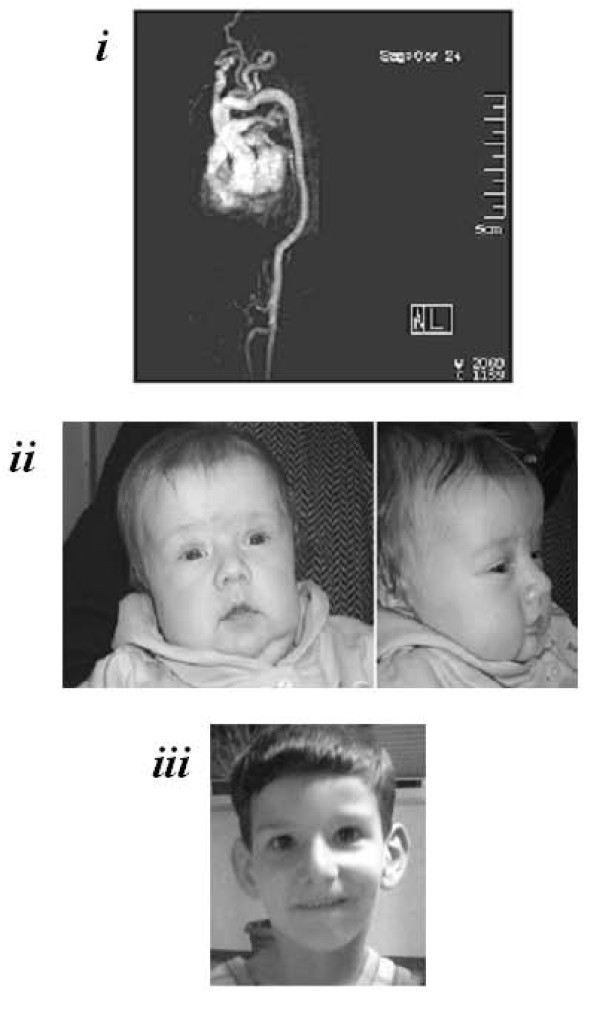
**Radiographic and facial features of the two Patients**. ***i) ***MRA of Patient 1 showed tortuosity and kinking of aortic arch, mild hypoplasia of descending and abdominal aorta, tortuosity of all the sovraortic trunks with kinking of left common carotid and left subclavian arteries, ectasia of the innominate artery origin, and bilateral reduction of peripheral pulmonary branches. ***ii) ***Patient 1 showed elongated face, micrognathia, mild blepharophimosis, downslanting palpebral fissures, beaked nose, high-arched palate. ***iii) ***Patient 2 showed elongated face, low-set and anteverted ears, down-slanting palpebral fissures, high nasal bridge.

Patient 1, an eight-months-old male baby, is the second-born from non-consanguineous unaffected parents. He was born by cesarean section for malposition at the 36^th ^week of gestation after a pregnancy complicated by oligoidramnios. At birth, his weight was 2.680 g and Apgar index of 7 and 8 at 1 and 5 minutes, respectively. Because progressive signs of respiratory distress (*i.e*., tachypnea and SatO_2 _of 83%) were noted soon after birth and given the presence of crackles at lung auscultation, antibiotic therapy and continuous positive airway pressure were started. Subsequently, the patient required synchronized intermittent mandatory ventilation with a FiO_2 _of about 45%-50% and was extubated after 8 days. Chest radiography, cranial and abdominal ultrasounds were normal. Cardiac echography showed a normal heart anatomy, but the presence of pulmonary hypertension signs and marked tortuosity of aortic arch. Magnetic Resonance Angiography (MRA) was needed (Figure 1,i). So far, staturo-ponderal growth is normal and, except facial dysmorphisms (Figure 1,ii), no cutaneous or skeletal involvement is noted. At six months the patient underwent to hernioplasty for bilateral congenital scrotal-inguinal hernia without post-surgery complications. At 7 month the patient was hospitalized for bronchiolitis and respiratory instability.

Patient 2, a five-years-old child, is the first of two sons of healthy non-consanguineous parents of South Italy origins. He came to medical attention, without a previous significant clinical history, at four years, for sudden dyspnoea and asthenia during a usual activity. Echocardiography excluded heart disease and showed pulmonary hypertension; angiography disclosed severe stenoses, elongation, and tortuosity of pulmonary arteries branches, aortic arch, sovraortic trunks, and iliac arteries. Pulmonary hypertension treatment was carried out as previously reported [10]. Patient's extravascular findings were: facial features (Figure 1, iii), high-arched palate, scoliosis, *pectus excavatum*, and joints hypermobility. No cutaneous, ocular, or tegument involvement was observed.

**Figure 2:**
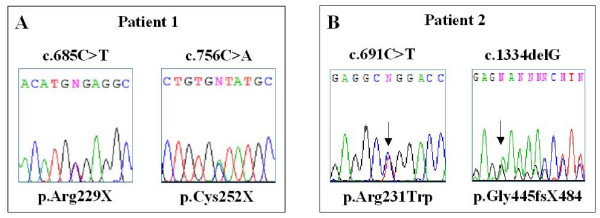
**Molecular characterization of the patients by *SLC2A10 *sequencing on genomic DNA, obtained from whole peripheral blood of the patients, after written informed consent given by the parents**. **A) **Patient 1 genotyping: the c.685C>T transition and the c.756C>A transversion, both in exon 2 and leading to p.Arg229X and the p.Cys252X nonsense mutations, respectively **B) **Chromatograms of mutations identified in Patient 2: the recurrent c.1334delG microdeletion in exon 3 and the novel c.691C>T transition in exon 2.

On the basis of the patients' phenotype *SLC2A10 *gene analysis was performed by exons amplification and direct sequencing, after written informed consent from patients parents. In Patient 1 genotyping disclosed the maternal c.685C>T transition and the paternal c.756C>A transversion, leading to p.Arg229X and the p.Cys252X nonsense mutations, respectively (Figure 2A). Both nucleotide substitutions were previously reported in homozygosity: the c.685C>T in a Belgian family [4], and the c.756C>A in a Kurdish patient [3]. In Patient 2 the maternal c.1334delG microdeletion, leading to the p.G445fsX484 recurrent mutation and the novel c.691C>T transition were disclosed (Figure 2B). The c.691C>T transition, detected in heterozygosity in the father and in the unaffected younger brother of the patient, affected the first position of the codon replacing a hydrophilic arginine residue with the hydrophobic tryptophan residue (p.Arg231Trp). The p.Arg231 was previously found substituted by a Gln residue, as the consequence of the c.692G>A transition, in three Spanish affected siblings, in compound heterozygosity with the c.1334delG mutation, like in our patient [4]. Nonsense and frameshift mutations, leading to a premature termination codon, could lead to nonsense mediated mRNA decay and allele loss-of-function [5]. On the other hand, missense mutations localized at an endofacial loop of GLUT10, like the p.Arg231Trp, should exert interference on the conformational changes of the protein necessary for the sugars transport [5,7].

## Conclusion

Two novel ATS patients were characterized at clinical and molecular level. Overall, four ATS unrelated families are known in Italy so far [[1,6,7,9], this work]. Of the 17 *SLC2A10 *mutations so far identified, 9 were missense, 4 nonsense and 4 small out-of-frame deletions leading to premature termination codons. The c.1334delG and the c.1309G>A mutations are recurrent, as well as the c.243C>G mutation found in Qatari [11] and Middle Eastern families [4,5,8]. The mutations here identified underline the recurrence of known *SLC2A10 *mutations among ATS patients, suggesting that these mutations arose more than one time, or likely should be referred to a founder effect, as shown for the c.1334delG deletion, already found in ATS patients from European countries and showing identical haplotype [4,5]. Among these, of note, both Italian families were from South Italy [[5], this work].

On the basis of previous observations and on the reports of clinical findings of patients carrying the same mutations of ours, no genotype-phenotype correlation can be drawn. Indeed, neither the type nor the position of mutations correlates with specific manifestations, or with type and onset of complications. In the patients here reported, the involvement of major- and medium-sized arteries was characteristic, though the extravascular manifestations were mild and not pathognomic for the disorder. Both patients were suspected as affected with ATS following the onset of acute respiratory symptoms, caused by hypertension of pulmonary branches, in the immediate perinatal period (Patient 1), and in the second infancy, in an apparent otherwise healthy child without a significant clinical history addressing a precocious diagnosis (Patient 2). Both conditions were life-threatening events needing emergency treatments and investigations. Though ATS prognosis has been recently downsized and thought to be less severe than initially reported (*i.e*., a mortality rate up to 40% before the age of 5 years), death in young age was referred to respiratory insufficiency, ventricular hypertrophy resulting in global heart failure, myocarditis, and ischemic events leading to organs infarction [2,4]. Among these causes, pulmonary infections, like the reported bronchiolitis in Patient 1, playing as trigger events of respiratory distress and acute cardiac failure, are thought to be serious events in young ATS patients [2,12-14].

Our results indicate that patients exhibiting arterial tortuosity with mild or without extravascular connective tissue manifestations should be screened for *SLC2A10 *gene mutations.

Further works in the comprehension of the natural history and on the molecular basis of ATS are needed to add new insights for diagnosis and prevention strategies for related complications.

## Consent

Written informed consent was obtained from patients parents for publication of this case report and accompanying images.

## List of abbreviations used

ATS: arterial tortuosity syndrome; *SLC2A10*: solute carrier family 2, member 10; GLUT10: facilitative glucose transporter 10; MRA: Magnetic Resonance Angiography.

## Competing interests

The authors declare that they have no competing interests.

## Authors' contributions

MC conceived the study. BD and MR carried out the molecular analysis, researched the literature reviewed and prepared the manuscript. BD, MV, GP, PB, MP, MAP diagnosed the patients. SB contributed to the discussion section. MC edited and coordinated the manuscript. All authors read and approved the final manuscript.
